# Crystal structure of {2-[({2-[(2-amino­ethyl)amino]­ethyl}imino)­meth­yl]-6-hy­droxy­phenolato-κ^4^ *N*,*N*′,*N*′′,*O* ^1^}(nitrato-κ*O*)copper(II) ethanol 0.25-solvate

**DOI:** 10.1107/S205698901501960X

**Published:** 2015-10-24

**Authors:** Shabana Noor, Sarvendra Kumar, Suhail Sabir, Rüdiger W. Seidel, Richard Goddard

**Affiliations:** a Department of Chemistry, Aligarh Muslim University, Aligarh, 202 002, India; b Faculty of Pharmaceutical Science, Tokyo University of Science, Noda, Japan; c Max-Planck-Institut für Kohlenforschung, Kaiser-Wilhelm-Platz-1, 45470 Mülheim an der Ruhr, Germany

**Keywords:** crystal structure, Cu^II^ complex, distorted square-pyramidal configuration, N—H⋯O hydrogen bond

## Abstract

In the crystal structure of the title mononuclear Cu^II^ complex, [Cu(C_11_H_16_N_3_O_2_)(NO_3_)]·0.25C_2_H_5_OH, the complex molecules are linked by N—H⋯O and O—H⋯O hydrogen bonds, forming a dimer with an approximate non-crystallographic twofold rotation axis of symmetry. In the monomeric unit, the Cu^2+^ ion exhibits a distorted square-pyramidal configuration, whereby the anionic [H*L*]^−^ Schiff base ligand binds in a tetradentate fashion *via* the O and the three N atoms which all are approximately coplanar. The O atom of a nitrate anion occupies the fifth coordination site, causing the Cu^II^ atom to move slightly out of the approximate basal plane toward the bound nitrate group. The structure exhibits disorder of the ethanol solvent mol­ecule.

## Related literature   

For the corresponding Schiff base, see: Osterbere (1974 ▸); Patterson & Holm (1975 ▸).
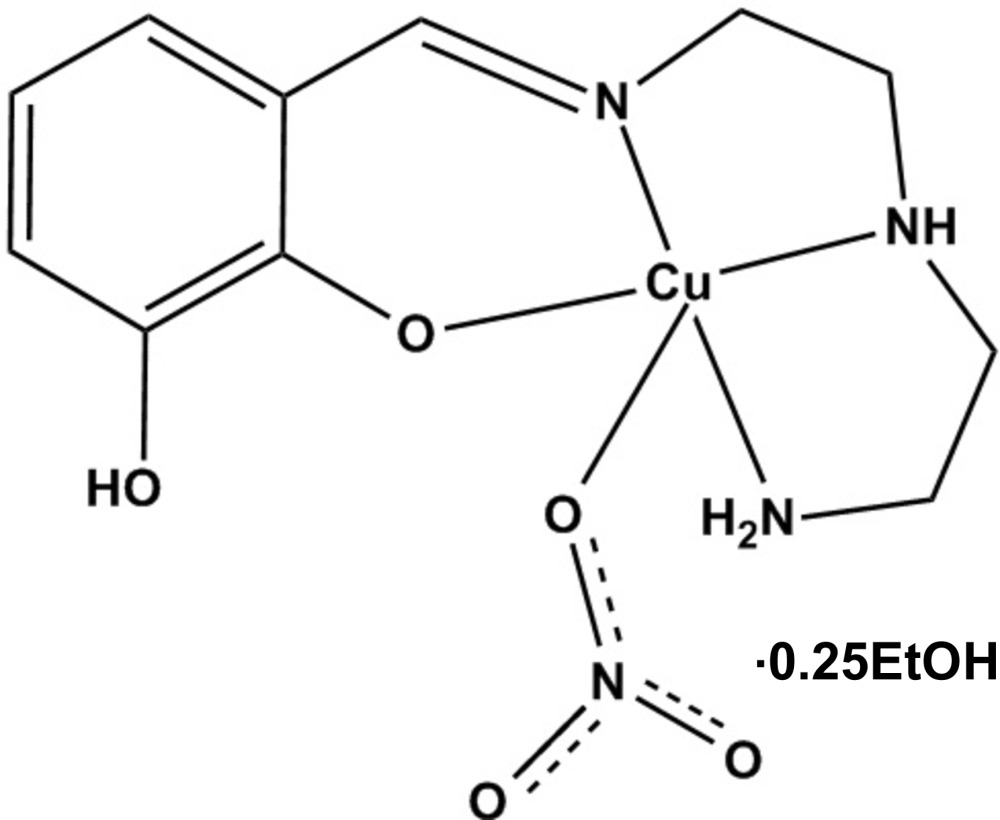


## Experimental   

### Crystal data  

[Cu(C_11_H_16_N_3_O_2_)(NO_3_)]·0.25C_2_H_6_O
*M* *_r_* = 359.33
Monoclinic, 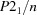

*a* = 11.952 (2) Å
*b* = 12.129 (2) Å
*c* = 19.590 (4) Å
β = 98.921 (3)°
*V* = 2805.5 (9) Å^3^
*Z* = 8
Mo *K*α radiation
μ = 1.59 mm^−1^
*T* = 100 K
0.16 × 0.11 × 0.08 mm

### Data collection  

Bruker AXS KappaCCD diffractometer
Absorption correction: Gaussian (*SADABS*; Bruker, 2013 ▸) *T*_min_ = 0.805, *T*_max_ = 0.879
106043 measured reflections
14120 independent reflections
10834 reflections with *I* > 2σ(*I*)
*R*_int_ = 0.059

### Refinement  

*R*[*F* ^2^ > 2σ(*F* ^2^)] = 0.044
*wR*(*F* ^2^) = 0.112
*S* = 1.07
14120 reflections
406 parameters
29 restraints
H-atom parameters constrained
Δρ_max_ = 1.06 e Å^−3^
Δρ_min_ = −1.15 e Å^−3^

### 

Data collection: *SMART* (Bruker, 2013 ▸); cell refinement: *SAINT* (Bruker, 2013 ▸); data reduction: *SAINT*; program(s) used to solve structure: *SHELXS2013* (Sheldrick, 2008 ▸); program(s) used to refine structure: *SHELXL2014* (Sheldrick, 2015 ▸); molecular graphics: *DIAMOND* (Brandenburg, & Berndt, 1999 ▸) and *Mercury* (Macrae *et al.*, 2008 ▸); software used to prepare material for publication: *enCIFer* (Allen *et al.*, 2004 ▸).

## Supplementary Material

Crystal structure: contains datablock(s) I, New_Global_Publ_Block. DOI: 10.1107/S205698901501960X/gw2154sup1.cif

Structure factors: contains datablock(s) I. DOI: 10.1107/S205698901501960X/gw2154Isup2.hkl

**Fig. 1.**
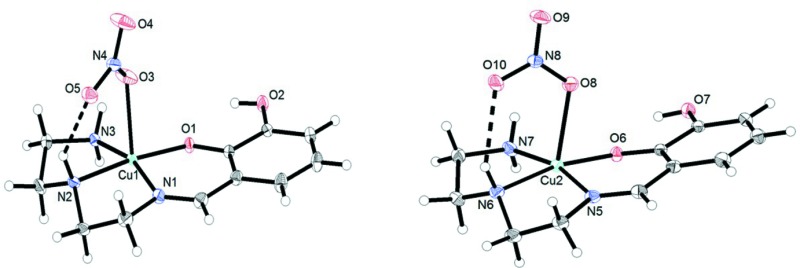
Crystal structure of title complex, [Cu(HL)(NO3)]·0.25EtOH, showing the two independent molecules in the crystal in similar orientations with labelling of significant atoms (Solvent is omitted for clarity).

L 3 . DOI: 10.1107/S205698901501960X/gw2154fig1.tif
Crystal structure of title complex, [Cu(H*L*)(NO_3_)]·0.25EtOH, showing the two independent mol­ecules in the crystal in similar orientations with labelling of significant atoms (Solvent is omitted for clarity).

**Fig. 2.**
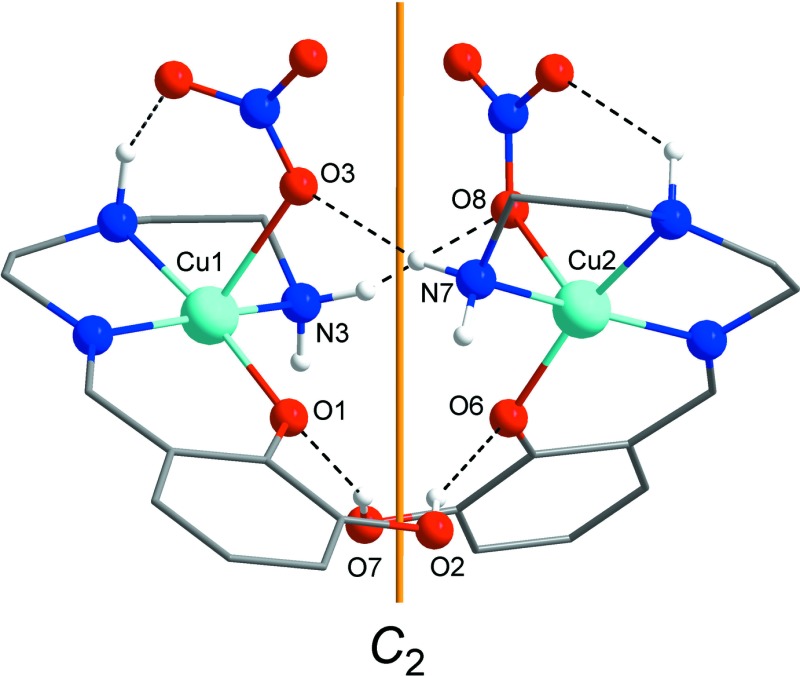
Dimer of the title complex, [Cu(HL)(NO3)]·0.25EtOH, showing the N—H···O and O—H···O hydrogen-bonding interactions. The approximate non-crystallographic 2-fold axis of symmetry of the dimer in the crystal is vertical. Selected distances (Å): N3···O8 2.931 (2), N7···O3 3.126 (2), O1···O7 2.697 (2), O2···O6 2.737 (2). Carbon-bound hydrogen atoms have been omitted for clarity.

L 3 . DOI: 10.1107/S205698901501960X/gw2154fig2.tif
Dimer of title complex, [Cu(H*L*)(NO_3_)]·0.25EtOH showing the N—H⋯O and O—H⋯O hydrogen-bonding inter­actions. The approximate non-crystallographic 2-fold axis of symmetry of the dimer in the crystal is vertical. Selected distances (Å): N3⋯O8 2.931 (2), N7⋯O3 3.126 (2), O1⋯O7 2.697 (2), O2⋯O6 2.737 (2). Carbon-bound hydrogen atoms have been omitted for clarity.

**Fig. 3.**
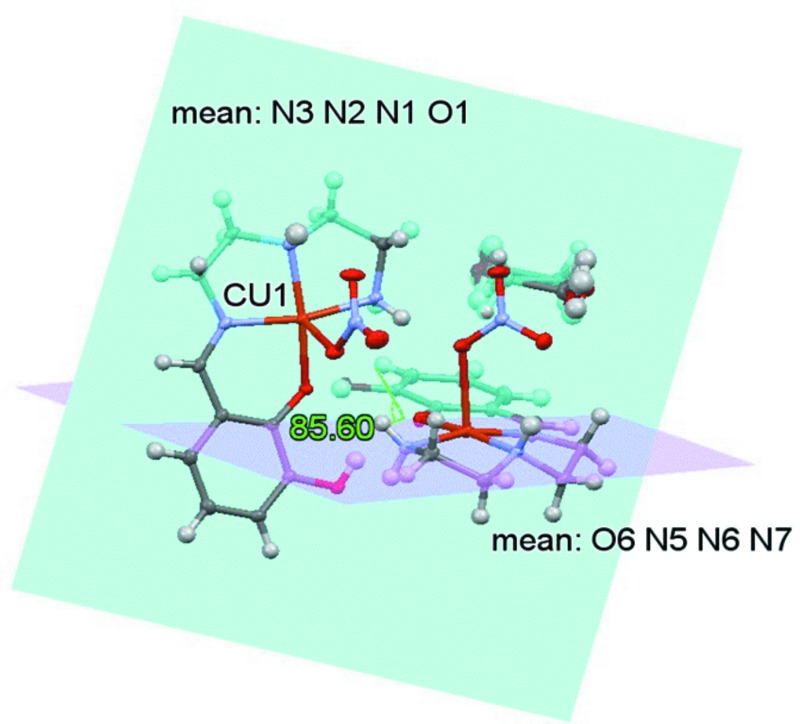
Arrangement of atoms in dimers of title complex, [Cu(HL)(NO3)]·0.25EtOH, in the crystal, showing the relationship between the approximate coordination planes of the Cu atoms defined by the coordinating N and O atoms (angle between the mean planes in °).

L 3 . DOI: 10.1107/S205698901501960X/gw2154fig3.tif
Arrangement of atoms in dimers of title complex, [Cu(H*L*)(NO_3_)]·0.25EtOH in the crystal, showing the relationship between the approximate coordination planes of the Cu atoms defined by the coordinating N and O atoms (angle between the mean planes in °).

CCDC reference: 1410216

Additional supporting information:  crystallographic information; 3D view; checkCIF report

## Figures and Tables

**Table 1:** Hydrogen-bond geometry (, )

*D*H*A*	*D*H	H*A*	*D* *A*	*D*H*A*
O2H2O1	0.84	2.28	2.7243(18)	114
O2H2O6	0.84	1.93	2.6969(18)	151
N2H2*A*O5	1.00	2.13	2.968(2)	140
N2H2*A*O10^i^	1.00	2.29	3.017(2)	129
N3H3*A*O8	0.91	2.25	3.126(2)	162
N3H3*B*O2^ii^	0.91	2.36	3.138(2)	143
N3H3*B*O7	0.91	2.47	3.071(2)	124
O7H7*A*O1	0.84	1.98	2.7365(18)	150
O7H7*A*O6	0.84	2.26	2.7096(18)	114
N6H6O5^iii^	1.00	2.10	2.960(2)	143
N6H6O10	1.00	2.34	3.067(2)	129
N7H7*B*O3	0.91	2.04	2.932(2)	167

Symmetry codes: (i) 
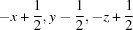
; (ii) 
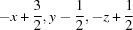
; (iii) 
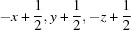
.

## supplementary crystallographic information

### S1. Structural commentary

Schiff base ligands and their metal complexes have been the subject of research covering a vast area of metallo–organic as well as bio–inorganic chemistry (Osterbere, 1974; Patterson & Holm, 1975). The characteristic features of the coordination behaviour of metal ions with Schiff base ligands make the complexes useful in variety of catalytic transformations. Tetra­nuclear manganese clusters with alkoxo bridges have been proved as potential models to elaborate the mechanism of oxygen evolution by polypeptides in the oxidation of water in photosynthetic organisms.

The title compound crystallizes in the monoclinic space group *P*2_1_/n with two crystallographic independent molecules of the complex, which differ essentially in the orientation of the nitrate group (Fig. 1). The complexes are linked by N—H···O and O—H···O hydrogen bonds to form a dimer with an approximate non-crystallographic 2-fold axis of symmetry, passing along a rough line described by the midpoints of Cu1 and Cu2, O3 and O8, N3 and N7, O1 and O6, and O2 and O7 (Fig. 2). In each monomer unit, the Cu^2+^ ion exhibits a distorted square-pyramidal configuration, whereby the anionic [HL] binds in tetra­dentate fashion via O and the three N atoms of the Schiff base ligand, which are approximately coplanar. The O atom of a nitrate anion occupies the fifth coordination site, causing the Cu atom to move slightly out of the approximate basal plane toward the bound nitrate group. For Cu1, this displacement is 0.167 Å above mean plane defined by O1, N1, N2 and N3, and for Cu2 it is 0.192 Å above the mean plane defined by O6, N5, N6 and N7. The Cu—N bond lengths (1.95-2.02 Å) are comparable with those of other structurally similar copper(II) complexes (1.95-2.28 Å). The Cu—O bond length to the apical O atom of the nitrate group at 2.36 (4) Å (mean) is significantly longer that the Cu—O bond length to the Schiff base ligand in the basal position of square pyramid [1.929 (1) Å (mean)]. The basal coordination planes of each Cu^II^ unit lie approximately perpendicular to one other (ca. 86^ο^) in the dimer, but this is not mirrored by the nitrate groups. The trigonality index τ values for the coordination spheres of the two independent Cu atoms are 0.16 and 0.03 [*τ* = (*β-α*)/60] where α and β are given by the main opposing angles in the coordination polyhedron (Fig.3). For perfect square pyramidal and trigonal bipyramidal coordination geometries, the values of *τ* are zero and unity, respectively. For Cu1 *β* = O(1)–Cu(1)–N(2) = 173.40 (6)° and *α* = N(1)–Cu(1)–N(3) = 163.61 (6)°] (Table 2). According to these values, the coordination geometry around both copper ions is best described as distorted square-pyramidal with one nitrate occupying the axial position. The relationship of coordination geometries of the Cu atoms of the monomer units in the dimer is shown in Fig. 3. Several of the NH groups of the ligands in the dimers are additionally involved in N—H···O hydrogen bonds to neighboring dimers [N3···O2 = 3.138 (2) Å, N6···O5 = 2.960 (2) Å, N2···O10 = 3.017 (2) Å].

### S2. Synthesis and crystallization

To a stirred solution of H_2_L (0.4 mmol, 0.089 g) dissolved in 40 mL of EtOH/CH_3_CN (1:1), Cu(NO_3_)_2_·3H_2_O (0.4 mmol, 0.096 g) was added, followed by addition of Et_3_N (0.12 mmol, 0.16 mL). The resulting mixture was refluxed for 5–6 h. The reaction mixture was filtered. After 2-5 d, dark-green crystals were obtained by slow diffusion of di­ethyl ether into the solution. IR data (KBr cm^-1^): 1613 ν(-CH=N), 3231, 3252, 1443 ν(N–H), 1223 ν(Ar–OH), 1110 ν(C–O) _,_585 ν(M–O), 540 ν(M–N).

### S3. Refinement

The ethanol solvent molecule is disordered about a centre of symmetry and was thus refined with site occupancy factors of 0.5. The O11—C23 and C23—C24 distances were restrained to target values of 1.43 (4) and 1.54 (4) Å, respectively. Rigid bond restraints and restraints towards isotropy were applied to the anisotropic atomic displacement parameters. H atoms were added at geometrically calculated positions and refined with the appropriate riding model.

### Crystal data

**Table:**

[Cu(C_11_H_16_N_3_O_2_)(NO_3_)]·0.25C_2_H_6_O	*F*(000) = 1484
*M**_r_* = 359.33	*D*_x_ = 1.701 Mg m^−^^3^
Monoclinic, *P*2_1_/*n*	Mo *K*α radiation, λ = 0.71073 Å
*a* = 11.952 (2) Å	Cell parameters from 10834 reflections
*b* = 12.129 (2) Å	θ = 0.8–0.9°
*c* = 19.590 (4) Å	µ = 1.59 mm^−^^1^
β = 98.921 (3)°	*T* = 100 K
*V* = 2805.5 (9) Å^3^	Prism, green
*Z* = 8	0.16 × 0.11 × 0.08 mm

### Data collection

**Table:**

Bruker AXS KappaCCD diffractometer	10834 reflections with *I* > 2σ(*I*)
Radiation source: FR591 rotating anode	*R*_int_ = 0.059
phi and ω scans	θ_max_ = 37.4°, θ_min_ = 3.0°
Absorption correction: multi-scan (*SADABS*; Bruker, 2013)	*h* = −20→20
*T*_min_ = 0.805, *T*_max_ = 0.879	*k* = −20→20
106043 measured reflections	*l* = −33→33
14120 independent reflections	

### Refinement

**Table:**

Refinement on *F*^2^	29 restraints
Least-squares matrix: full	Hydrogen site location: inferred from neighbouring sites
*R*[*F*^2^ > 2σ(*F*^2^)] = 0.044	H-atom parameters constrained
*wR*(*F*^2^) = 0.112	*w* = 1/[σ^2^(*F*_o_^2^) + (0.0392*P*)^2^ + 3.8944*P*] where *P* = (*F*_o_^2^ + 2*F*_c_^2^)/3
*S* = 1.07	(Δ/σ)_max_ = 0.001
14120 reflections	Δρ_max_ = 1.06 e Å^−^^3^
406 parameters	Δρ_min_ = −1.15 e Å^−^^3^

### Special details

**Table:**

Geometry. All e.s.d.'s (except the e.s.d. in the dihedral angle between two l.s. planes) are estimated using the full covariance matrix. The cell e.s.d.'s are taken into account individually in the estimation of e.s.d.'s in distances, angles and torsion angles; correlations between e.s.d.'s in cell parameters are only used when they are defined by crystal symmetry. An approximate (isotropic) treatment of cell e.s.d.'s is used for estimating e.s.d.'s involving l.s. planes.

### Fractional atomic coordinates and isotropic or equivalent isotropic displacement parameters (Å^2^)

**Table:**

	*x*	*y*	*z*	*U*_iso_*/*U*_eq_	Occ. (<1)
Cu1	0.56242 (2)	0.22501 (2)	0.31990 (2)	0.01125 (4)	
O1	0.67530 (10)	0.33955 (10)	0.32549 (6)	0.0133 (2)	
O2	0.79704 (11)	0.52715 (11)	0.31545 (7)	0.0178 (2)	
H2	0.7585	0.4893	0.2844	0.027*	
O3	0.41682 (13)	0.35390 (12)	0.31382 (9)	0.0280 (3)	
O4	0.27794 (16)	0.43188 (14)	0.35387 (11)	0.0373 (4)	
O5	0.30557 (12)	0.25521 (12)	0.36722 (8)	0.0217 (3)	
N1	0.59587 (12)	0.19043 (13)	0.41797 (7)	0.0149 (2)	
N2	0.46006 (12)	0.09379 (12)	0.31626 (8)	0.0140 (2)	
H2A	0.3833	0.1202	0.3231	0.017*	
N3	0.53736 (12)	0.21467 (12)	0.21599 (8)	0.0135 (2)	
H3A	0.5207	0.2823	0.1971	0.016*	
H3B	0.6010	0.1892	0.2011	0.016*	
N4	0.33393 (13)	0.34802 (13)	0.34611 (8)	0.0166 (3)	
C1	0.72842 (13)	0.38286 (14)	0.38339 (8)	0.0124 (2)	
C2	0.79243 (14)	0.48064 (14)	0.37829 (9)	0.0139 (3)	
C3	0.85100 (15)	0.53096 (15)	0.43647 (9)	0.0183 (3)	
H3	0.8931	0.5962	0.4316	0.022*	
C4	0.84897 (17)	0.48710 (18)	0.50225 (10)	0.0222 (4)	
H4	0.8896	0.5221	0.5419	0.027*	
C5	0.78789 (17)	0.39301 (18)	0.50912 (9)	0.0207 (3)	
H5	0.7868	0.3630	0.5538	0.025*	
C6	0.72649 (14)	0.34007 (15)	0.45065 (9)	0.0152 (3)	
C7	0.66276 (15)	0.24331 (16)	0.46398 (9)	0.0174 (3)	
H7	0.6711	0.2171	0.5102	0.021*	
C8	0.53173 (16)	0.09540 (16)	0.43800 (10)	0.0188 (3)	
H8A	0.4611	0.1207	0.4537	0.023*	
H8B	0.5775	0.0546	0.4763	0.023*	
C9	0.50393 (15)	0.02128 (14)	0.37510 (10)	0.0179 (3)	
H9A	0.5727	−0.0180	0.3659	0.021*	
H9B	0.4462	−0.0340	0.3827	0.021*	
C10	0.44999 (16)	0.04397 (15)	0.24709 (10)	0.0186 (3)	
H10A	0.3816	−0.0030	0.2382	0.022*	
H10B	0.5171	−0.0023	0.2435	0.022*	
C11	0.44169 (15)	0.13761 (15)	0.19500 (9)	0.0179 (3)	
H11A	0.4453	0.1080	0.1483	0.022*	
H11B	0.3687	0.1768	0.1937	0.022*	
Cu2	0.53711 (2)	0.58282 (2)	0.19038 (2)	0.01359 (5)	
O6	0.66003 (10)	0.47838 (11)	0.19655 (6)	0.0144 (2)	
O7	0.78265 (11)	0.28994 (11)	0.21571 (7)	0.0164 (2)	
H7A	0.7543	0.3288	0.2442	0.025*	
O8	0.42326 (11)	0.42812 (12)	0.14803 (8)	0.0209 (3)	
O9	0.25873 (13)	0.34741 (13)	0.13124 (9)	0.0269 (3)	
O10	0.27202 (12)	0.51948 (13)	0.16051 (9)	0.0273 (3)	
N5	0.56098 (12)	0.63534 (12)	0.09942 (8)	0.0154 (2)	
N6	0.43573 (13)	0.71478 (13)	0.18807 (8)	0.0164 (3)	
H6	0.3579	0.6929	0.1659	0.020*	
N7	0.51519 (14)	0.56904 (13)	0.28940 (8)	0.0182 (3)	
H7B	0.4958	0.4986	0.2984	0.022*	
H7C	0.5808	0.5858	0.3177	0.022*	
N8	0.31697 (13)	0.43119 (12)	0.14697 (8)	0.0152 (2)	
C12	0.69497 (13)	0.43548 (13)	0.14185 (8)	0.0125 (3)	
C13	0.75845 (13)	0.33588 (14)	0.15149 (9)	0.0136 (3)	
C14	0.79762 (15)	0.28423 (14)	0.09677 (9)	0.0165 (3)	
H14	0.8388	0.2172	0.1044	0.020*	
C15	0.77738 (16)	0.32950 (16)	0.03034 (9)	0.0193 (3)	
H15	0.8043	0.2934	−0.0070	0.023*	
C16	0.71802 (16)	0.42696 (16)	0.01954 (9)	0.0191 (3)	
H16	0.7056	0.4586	−0.0254	0.023*	
C17	0.67533 (15)	0.48069 (14)	0.07403 (9)	0.0154 (3)	
C18	0.61363 (15)	0.58271 (15)	0.05751 (9)	0.0164 (3)	
H18	0.6122	0.6125	0.0125	0.020*	
C19	0.50038 (15)	0.73849 (15)	0.07816 (10)	0.0179 (3)	
H19A	0.4275	0.7223	0.0484	0.021*	
H19B	0.5464	0.7854	0.0519	0.021*	
C20	0.47980 (16)	0.79697 (15)	0.14363 (10)	0.0197 (3)	
H20A	0.5514	0.8290	0.1677	0.024*	
H20B	0.4243	0.8573	0.1322	0.024*	
C21	0.43072 (17)	0.74907 (15)	0.25968 (10)	0.0195 (3)	
H21A	0.3632	0.7958	0.2612	0.023*	
H21B	0.4991	0.7922	0.2782	0.023*	
C22	0.42422 (17)	0.64578 (16)	0.30250 (10)	0.0200 (3)	
H22A	0.4339	0.6650	0.3522	0.024*	
H22B	0.3493	0.6103	0.2897	0.024*	
O11	0.5614 (5)	0.6050 (5)	0.4859 (4)	0.075 (2)	0.5
H11	0.5313	0.6421	0.5144	0.113*	0.5
C23	0.4943 (5)	0.5153 (5)	0.4647 (3)	0.0330 (10)	0.5
H23A	0.5303	0.4739	0.4303	0.040*	0.5
H23B	0.4205	0.5428	0.4408	0.040*	0.5
C24	0.4721 (6)	0.4375 (5)	0.5187 (3)	0.0381 (12)	0.5
H24A	0.4237	0.3775	0.4976	0.057*	0.5
H24B	0.5439	0.4071	0.5419	0.057*	0.5
H24C	0.4338	0.4761	0.5524	0.057*	0.5

### Atomic displacement parameters (Å^2^)

**Table:**

	*U*^11^	*U*^22^	*U*^33^	*U*^12^	*U*^13^	*U*^23^
Cu1	0.01251 (8)	0.00876 (8)	0.01241 (8)	−0.00203 (6)	0.00167 (6)	0.00106 (6)
O1	0.0144 (5)	0.0132 (5)	0.0120 (5)	−0.0049 (4)	0.0007 (4)	0.0001 (4)
O2	0.0200 (6)	0.0154 (6)	0.0166 (5)	−0.0066 (5)	−0.0012 (4)	0.0027 (4)
O3	0.0268 (7)	0.0157 (6)	0.0470 (9)	0.0056 (5)	0.0229 (7)	0.0088 (6)
O4	0.0363 (9)	0.0180 (7)	0.0643 (12)	0.0118 (6)	0.0285 (9)	0.0078 (7)
O5	0.0204 (6)	0.0168 (6)	0.0290 (7)	−0.0020 (5)	0.0071 (5)	0.0048 (5)
N1	0.0159 (6)	0.0139 (6)	0.0152 (6)	−0.0031 (5)	0.0030 (5)	0.0033 (5)
N2	0.0130 (5)	0.0095 (5)	0.0195 (6)	0.0006 (4)	0.0023 (5)	0.0017 (5)
N3	0.0130 (5)	0.0102 (5)	0.0171 (6)	−0.0010 (4)	0.0014 (4)	0.0004 (4)
N4	0.0158 (6)	0.0142 (6)	0.0199 (7)	0.0020 (5)	0.0035 (5)	0.0018 (5)
C1	0.0117 (6)	0.0120 (6)	0.0130 (6)	−0.0007 (5)	0.0004 (5)	−0.0010 (5)
C2	0.0132 (6)	0.0112 (6)	0.0166 (7)	−0.0008 (5)	0.0008 (5)	−0.0002 (5)
C3	0.0184 (7)	0.0157 (7)	0.0197 (7)	−0.0034 (6)	−0.0005 (6)	−0.0035 (6)
C4	0.0240 (8)	0.0250 (9)	0.0164 (7)	−0.0059 (7)	−0.0005 (6)	−0.0069 (6)
C5	0.0233 (8)	0.0256 (9)	0.0125 (7)	−0.0042 (7)	0.0000 (6)	−0.0030 (6)
C6	0.0158 (7)	0.0168 (7)	0.0129 (6)	−0.0008 (6)	0.0015 (5)	0.0005 (5)
C7	0.0180 (7)	0.0207 (8)	0.0133 (7)	−0.0008 (6)	0.0021 (5)	0.0038 (6)
C8	0.0178 (7)	0.0185 (8)	0.0204 (8)	−0.0027 (6)	0.0041 (6)	0.0083 (6)
C9	0.0149 (7)	0.0115 (7)	0.0269 (8)	−0.0010 (5)	0.0025 (6)	0.0065 (6)
C10	0.0190 (7)	0.0114 (7)	0.0252 (8)	−0.0034 (6)	0.0027 (6)	−0.0025 (6)
C11	0.0174 (7)	0.0158 (7)	0.0191 (7)	−0.0033 (6)	−0.0021 (6)	−0.0022 (6)
Cu2	0.01411 (9)	0.01017 (9)	0.01689 (9)	0.00239 (7)	0.00369 (7)	0.00333 (7)
O6	0.0146 (5)	0.0151 (5)	0.0137 (5)	0.0041 (4)	0.0029 (4)	0.0019 (4)
O7	0.0183 (5)	0.0145 (5)	0.0168 (5)	0.0039 (4)	0.0042 (4)	0.0036 (4)
O8	0.0142 (5)	0.0161 (6)	0.0325 (7)	0.0015 (4)	0.0041 (5)	0.0020 (5)
O9	0.0239 (7)	0.0171 (6)	0.0378 (8)	−0.0061 (5)	−0.0012 (6)	−0.0051 (6)
O10	0.0179 (6)	0.0173 (6)	0.0474 (9)	0.0026 (5)	0.0076 (6)	−0.0077 (6)
N5	0.0148 (6)	0.0118 (6)	0.0190 (6)	0.0004 (5)	0.0007 (5)	0.0042 (5)
N6	0.0143 (6)	0.0132 (6)	0.0219 (7)	0.0009 (5)	0.0033 (5)	0.0036 (5)
N7	0.0238 (7)	0.0117 (6)	0.0195 (7)	0.0028 (5)	0.0044 (5)	0.0010 (5)
N8	0.0158 (6)	0.0139 (6)	0.0153 (6)	−0.0009 (5)	0.0002 (5)	0.0014 (5)
C12	0.0121 (6)	0.0111 (6)	0.0144 (6)	−0.0006 (5)	0.0024 (5)	0.0006 (5)
C13	0.0124 (6)	0.0113 (6)	0.0170 (7)	−0.0010 (5)	0.0023 (5)	0.0005 (5)
C14	0.0176 (7)	0.0123 (7)	0.0201 (7)	0.0003 (5)	0.0042 (6)	−0.0018 (5)
C15	0.0225 (8)	0.0190 (8)	0.0170 (7)	−0.0019 (6)	0.0047 (6)	−0.0031 (6)
C16	0.0241 (8)	0.0186 (8)	0.0148 (7)	−0.0013 (6)	0.0036 (6)	0.0003 (6)
C17	0.0169 (7)	0.0135 (7)	0.0158 (7)	−0.0010 (5)	0.0024 (5)	0.0014 (5)
C18	0.0180 (7)	0.0148 (7)	0.0155 (7)	0.0003 (6)	0.0002 (5)	0.0041 (5)
C19	0.0176 (7)	0.0141 (7)	0.0211 (8)	0.0025 (6)	0.0006 (6)	0.0060 (6)
C20	0.0204 (8)	0.0116 (7)	0.0270 (9)	0.0017 (6)	0.0039 (6)	0.0052 (6)
C21	0.0221 (8)	0.0119 (7)	0.0250 (8)	0.0022 (6)	0.0054 (6)	−0.0001 (6)
C22	0.0229 (8)	0.0151 (7)	0.0239 (8)	0.0032 (6)	0.0094 (6)	0.0020 (6)
O11	0.073 (4)	0.051 (3)	0.091 (4)	−0.036 (3)	−0.019 (3)	0.022 (3)
C23	0.032 (2)	0.040 (3)	0.027 (2)	0.004 (2)	0.0071 (18)	0.0094 (19)
C24	0.043 (3)	0.029 (3)	0.044 (3)	−0.003 (2)	0.009 (3)	0.001 (2)

### Geometric parameters (Å, º)

**Table:**

Cu1—O1	1.9278 (12)	Cu2—O8	2.3896 (15)
Cu1—N1	1.9465 (15)	O6—C12	1.316 (2)
Cu1—N2	2.0020 (15)	O7—C13	1.365 (2)
Cu1—N3	2.0150 (15)	O7—H7A	0.8400
Cu1—O3	2.3286 (15)	O8—N8	1.268 (2)
O1—C1	1.3200 (19)	O9—N8	1.243 (2)
O2—C2	1.363 (2)	O10—N8	1.245 (2)
O2—H2	0.8400	N5—C18	1.280 (2)
O3—N4	1.257 (2)	N5—C19	1.473 (2)
O4—N4	1.240 (2)	N6—C21	1.473 (3)
O5—N4	1.264 (2)	N6—C20	1.473 (2)
N1—C7	1.280 (2)	N6—H6	1.0000
N1—C8	1.471 (2)	N7—C22	1.483 (2)
N2—C10	1.472 (2)	N7—H7B	0.9100
N2—C9	1.480 (2)	N7—H7C	0.9100
N2—H2A	1.0000	C12—C17	1.423 (2)
N3—C11	1.485 (2)	C12—C13	1.423 (2)
N3—H3A	0.9100	C13—C14	1.384 (2)
N3—H3B	0.9100	C14—C15	1.399 (3)
C1—C6	1.420 (2)	C14—H14	0.9500
C1—C2	1.423 (2)	C15—C16	1.378 (3)
C2—C3	1.383 (2)	C15—H15	0.9500
C3—C4	1.398 (3)	C16—C17	1.412 (3)
C3—H3	0.9500	C16—H16	0.9500
C4—C5	1.373 (3)	C17—C18	1.451 (2)
C4—H4	0.9500	C18—H18	0.9500
C5—C6	1.415 (2)	C19—C20	1.519 (3)
C5—H5	0.9500	C19—H19A	0.9900
C6—C7	1.445 (3)	C19—H19B	0.9900
C7—H7	0.9500	C20—H20A	0.9900
C8—C9	1.520 (3)	C20—H20B	0.9900
C8—H8A	0.9900	C21—C22	1.516 (3)
C8—H8B	0.9900	C21—H21A	0.9900
C9—H9A	0.9900	C21—H21B	0.9900
C9—H9B	0.9900	C22—H22A	0.9900
C10—C11	1.520 (3)	C22—H22B	0.9900
C10—H10A	0.9900	O11—C23	1.377 (8)
C10—H10B	0.9900	O11—H11	0.8400
C11—H11A	0.9900	C23—C24	1.471 (8)
C11—H11B	0.9900	C23—H23A	0.9900
Cu2—O6	1.9295 (12)	C23—H23B	0.9900
Cu2—N5	1.9545 (15)	C24—H24A	0.9800
Cu2—N6	2.0037 (15)	C24—H24B	0.9800
Cu2—N7	2.0043 (16)	C24—H24C	0.9800
			
O1—Cu1—N1	93.69 (6)	N5—Cu2—O8	95.46 (6)
O1—Cu1—N2	173.40 (6)	N6—Cu2—O8	108.03 (6)
N1—Cu1—N2	83.95 (6)	N7—Cu2—O8	96.76 (6)
O1—Cu1—N3	95.47 (5)	C12—O6—Cu2	122.90 (11)
N1—Cu1—N3	163.61 (6)	C13—O7—H7A	109.5
N2—Cu1—N3	85.43 (6)	N8—O8—Cu2	119.86 (11)
O1—Cu1—O3	91.72 (6)	C18—N5—C19	120.75 (15)
N1—Cu1—O3	103.45 (6)	C18—N5—Cu2	125.32 (12)
N2—Cu1—O3	94.83 (6)	C19—N5—Cu2	113.56 (12)
N3—Cu1—O3	89.85 (6)	C21—N6—C20	116.27 (15)
C1—O1—Cu1	125.08 (10)	C21—N6—Cu2	108.52 (11)
C2—O2—H2	109.5	C20—N6—Cu2	106.46 (11)
N4—O3—Cu1	125.24 (12)	C21—N6—H6	108.5
C7—N1—C8	119.95 (15)	C20—N6—H6	108.5
C7—N1—Cu1	126.66 (12)	Cu2—N6—H6	108.5
C8—N1—Cu1	113.35 (11)	C22—N7—Cu2	108.93 (12)
C10—N2—C9	116.22 (14)	C22—N7—H7B	109.9
C10—N2—Cu1	108.75 (11)	Cu2—N7—H7B	109.9
C9—N2—Cu1	107.70 (10)	C22—N7—H7C	109.9
C10—N2—H2A	108.0	Cu2—N7—H7C	109.9
C9—N2—H2A	108.0	H7B—N7—H7C	108.3
Cu1—N2—H2A	108.0	O9—N8—O10	120.82 (16)
C11—N3—Cu1	107.84 (11)	O9—N8—O8	120.08 (16)
C11—N3—H3A	110.1	O10—N8—O8	119.09 (15)
Cu1—N3—H3A	110.1	O6—C12—C17	125.50 (15)
C11—N3—H3B	110.1	O6—C12—C13	117.21 (14)
Cu1—N3—H3B	110.1	C17—C12—C13	117.29 (15)
H3A—N3—H3B	108.5	O7—C13—C14	118.69 (15)
O4—N4—O3	119.83 (16)	O7—C13—C12	120.07 (15)
O4—N4—O5	120.90 (16)	C14—C13—C12	121.23 (15)
O3—N4—O5	119.17 (15)	C13—C14—C15	120.82 (16)
O1—C1—C6	125.26 (15)	C13—C14—H14	119.6
O1—C1—C2	117.61 (14)	C15—C14—H14	119.6
C6—C1—C2	117.13 (14)	C16—C15—C14	119.37 (17)
O2—C2—C3	118.38 (15)	C16—C15—H15	120.3
O2—C2—C1	120.37 (14)	C14—C15—H15	120.3
C3—C2—C1	121.24 (16)	C15—C16—C17	121.17 (17)
C2—C3—C4	120.84 (17)	C15—C16—H16	119.4
C2—C3—H3	119.6	C17—C16—H16	119.4
C4—C3—H3	119.6	C16—C17—C12	120.10 (16)
C5—C4—C3	119.50 (17)	C16—C17—C18	117.12 (16)
C5—C4—H4	120.3	C12—C17—C18	122.78 (16)
C3—C4—H4	120.3	N5—C18—C17	124.45 (16)
C4—C5—C6	120.99 (17)	N5—C18—H18	117.8
C4—C5—H5	119.5	C17—C18—H18	117.8
C6—C5—H5	119.5	N5—C19—C20	107.15 (14)
C5—C6—C1	120.29 (16)	N5—C19—H19A	110.3
C5—C6—C7	116.37 (16)	C20—C19—H19A	110.3
C1—C6—C7	123.34 (15)	N5—C19—H19B	110.3
N1—C7—C6	124.67 (16)	C20—C19—H19B	110.3
N1—C7—H7	117.7	H19A—C19—H19B	108.5
C6—C7—H7	117.7	N6—C20—C19	107.61 (15)
N1—C8—C9	107.75 (14)	N6—C20—H20A	110.2
N1—C8—H8A	110.2	C19—C20—H20A	110.2
C9—C8—H8A	110.2	N6—C20—H20B	110.2
N1—C8—H8B	110.2	C19—C20—H20B	110.2
C9—C8—H8B	110.2	H20A—C20—H20B	108.5
H8A—C8—H8B	108.5	N6—C21—C22	107.86 (15)
N2—C9—C8	106.64 (14)	N6—C21—H21A	110.1
N2—C9—H9A	110.4	C22—C21—H21A	110.1
C8—C9—H9A	110.4	N6—C21—H21B	110.1
N2—C9—H9B	110.4	C22—C21—H21B	110.1
C8—C9—H9B	110.4	H21A—C21—H21B	108.4
H9A—C9—H9B	108.6	N7—C22—C21	108.72 (15)
N2—C10—C11	107.39 (14)	N7—C22—H22A	109.9
N2—C10—H10A	110.2	C21—C22—H22A	109.9
C11—C10—H10A	110.2	N7—C22—H22B	109.9
N2—C10—H10B	110.2	C21—C22—H22B	109.9
C11—C10—H10B	110.2	H22A—C22—H22B	108.3
H10A—C10—H10B	108.5	C23—O11—H11	109.5
N3—C11—C10	108.50 (14)	O11—C23—C24	116.9 (6)
N3—C11—H11A	110.0	O11—C23—H23A	108.1
C10—C11—H11A	110.0	C24—C23—H23A	108.1
N3—C11—H11B	110.0	O11—C23—H23B	108.1
C10—C11—H11B	110.0	C24—C23—H23B	108.1
H11A—C11—H11B	108.4	H23A—C23—H23B	107.3
O6—Cu2—N5	93.08 (6)	C23—C24—H24A	109.5
O6—Cu2—N6	167.90 (6)	C23—C24—H24B	109.5
N5—Cu2—N6	83.82 (6)	H24A—C24—H24B	109.5
O6—Cu2—N7	95.58 (6)	C23—C24—H24C	109.5
N5—Cu2—N7	165.70 (6)	H24A—C24—H24C	109.5
N6—Cu2—N7	85.36 (6)	H24B—C24—H24C	109.5
O6—Cu2—O8	83.87 (5)		

### Hydrogen-bond geometry (Å, º)

**Table:**

*D*—H···*A*	*D*—H	H···*A*	*D*···*A*	*D*—H···*A*
O2—H2···O1	0.84	2.28	2.7243 (18)	114
O2—H2···O6	0.84	1.93	2.6969 (18)	151
N2—H2*A*···O5	1.00	2.13	2.968 (2)	140
N2—H2*A*···O10^i^	1.00	2.29	3.017 (2)	129
N3—H3*A*···O8	0.91	2.25	3.126 (2)	162
N3—H3*B*···O2^ii^	0.91	2.36	3.138 (2)	143
N3—H3*B*···O7	0.91	2.47	3.071 (2)	124
O7—H7*A*···O1	0.84	1.98	2.7365 (18)	150
O7—H7*A*···O6	0.84	2.26	2.7096 (18)	114
N6—H6···O5^iii^	1.00	2.10	2.960 (2)	143
N6—H6···O10	1.00	2.34	3.067 (2)	129
N7—H7*B*···O3	0.91	2.04	2.932 (2)	167

Symmetry codes: (i) −*x*+1/2, *y*−1/2, −*z*+1/2; (ii) −*x*+3/2, *y*−1/2, −*z*+1/2; (iii) −*x*+1/2, *y*+1/2, −*z*+1/2.
