# Supplementary figures and images for: Crystal structure of {2-[({2-[(2-amino­ethyl)amino]­ethyl}imino)­meth­yl]-6-hy­droxy­phenolato-κ4 N,N′,N′′,O 1}(nitrato-κO)copper(II) ethanol 0.25-solvate
Source: Acta Crystallogr E Crystallogr Commun. 2015 Oct 24;71(Pt 11):m205–6. doi: 10.1107/S205698901501960X (PMC4645037; doi:10.1107/S205698901501960X)

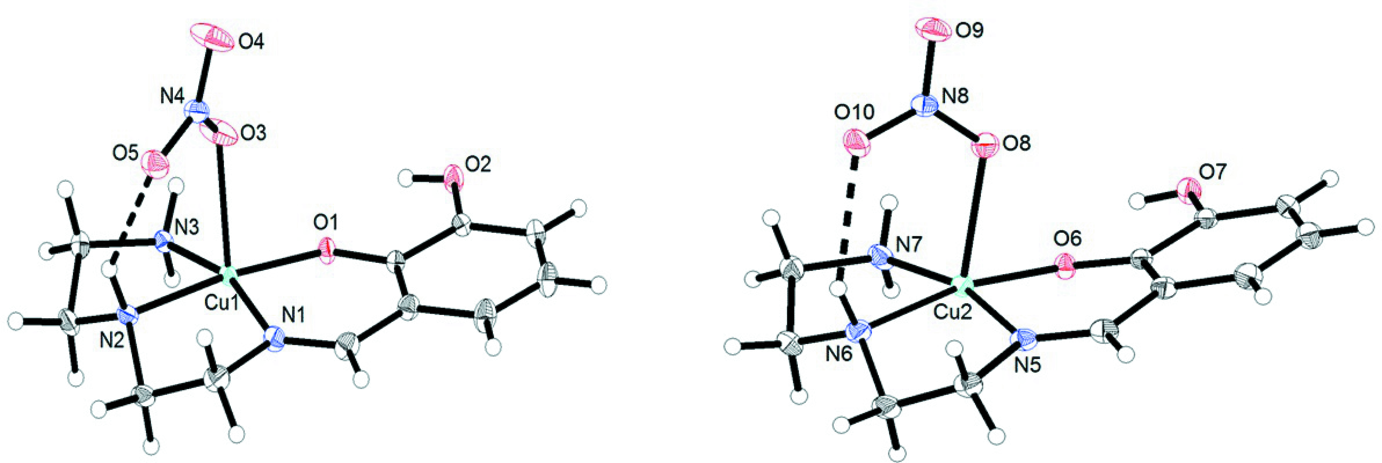

Supplement: Supplementary file 3 [file e-71-0m205-fig1.tif]

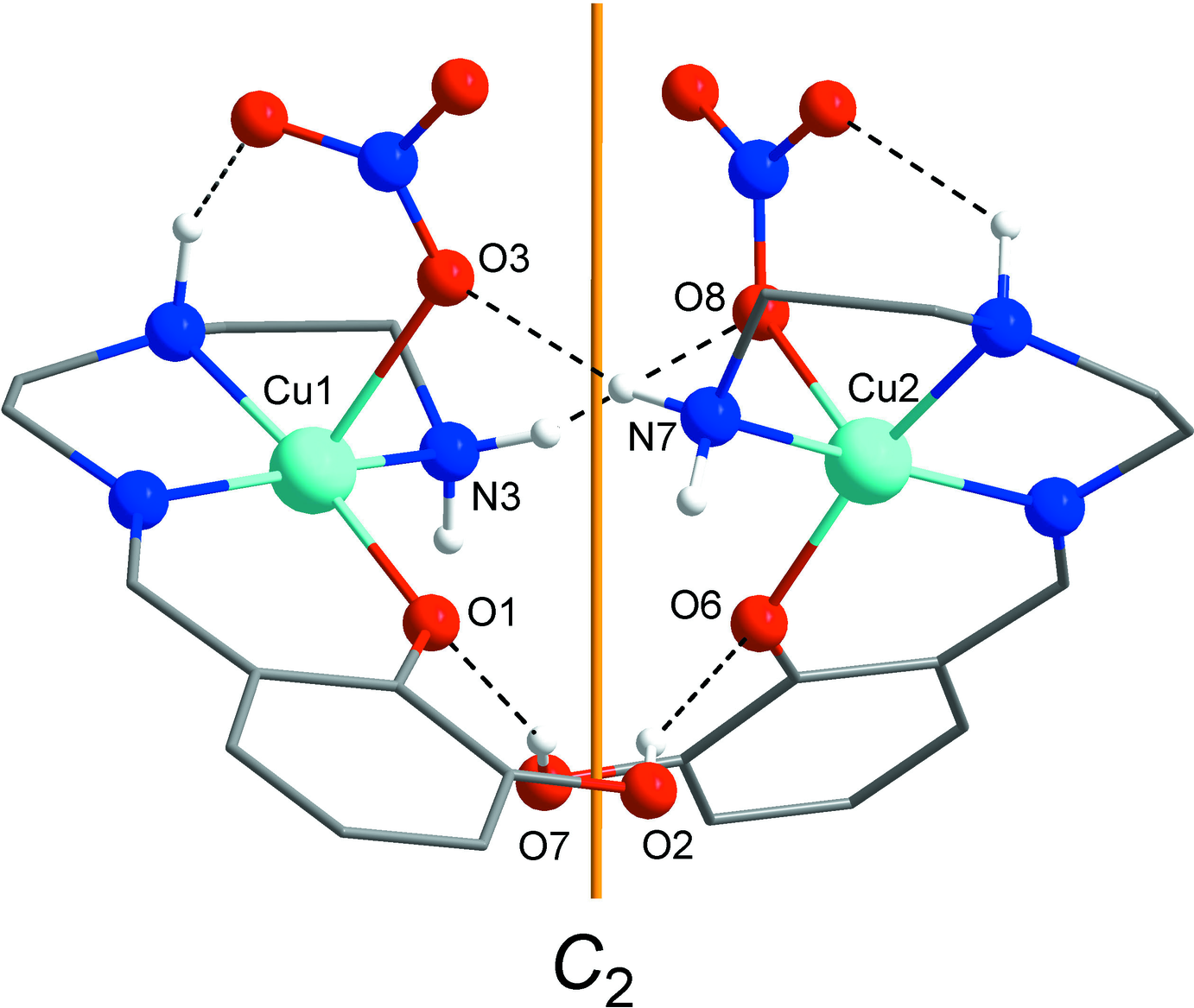

Supplement: Supplementary file 4 [file e-71-0m205-fig2.tif]

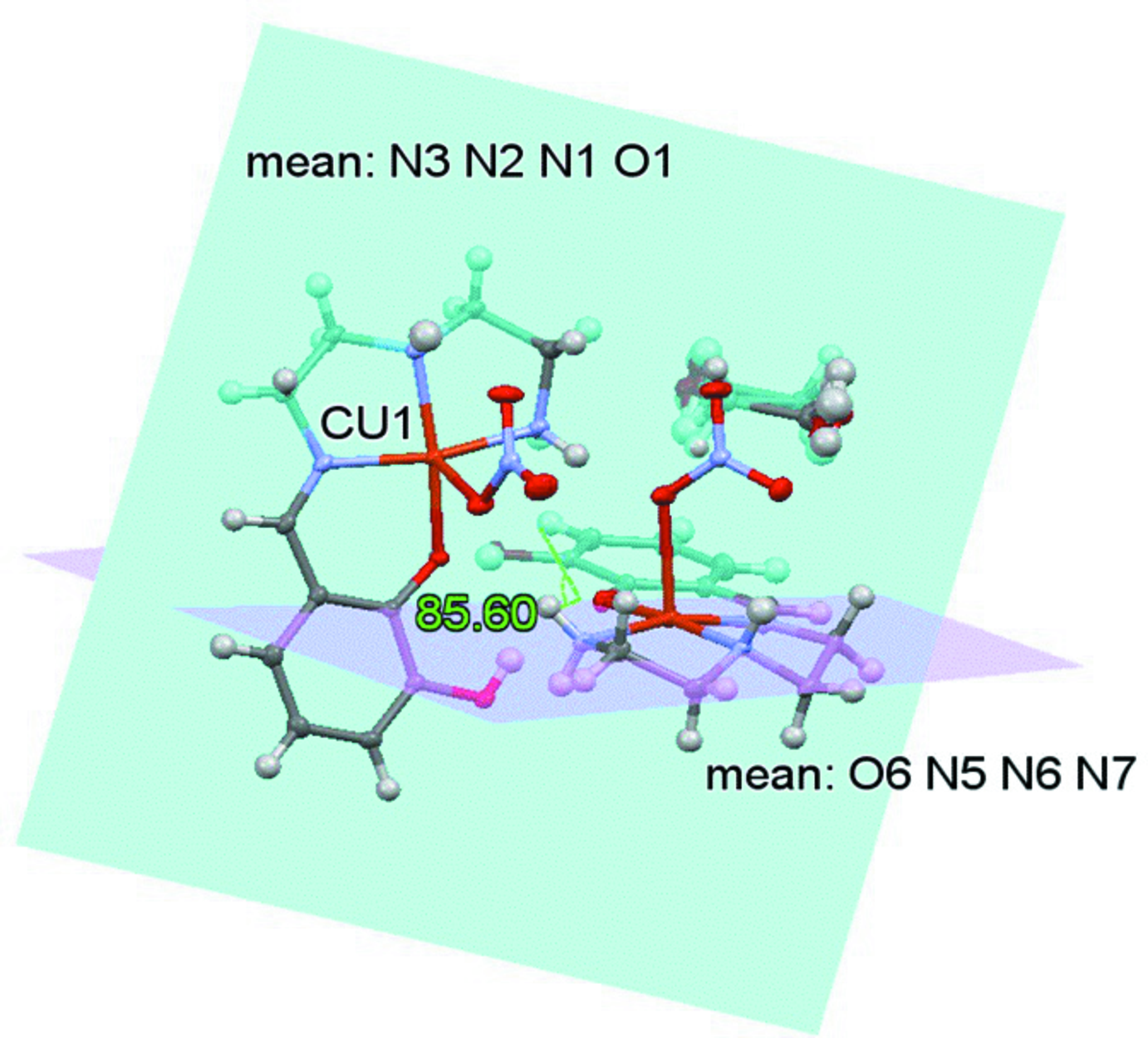

Supplement: Supplementary file 5 [file e-71-0m205-fig3.tif]
